# A Vision-Based Approach to UAV Detection and Tracking in Cooperative Applications

**DOI:** 10.3390/s18103391

**Published:** 2018-10-10

**Authors:** Roberto Opromolla, Giancarmine Fasano, Domenico Accardo

**Affiliations:** Department of Industrial Engineering, University of Naples Federico II, Piazzale Tecchio 80, 80125 Naples, Italy; giancarmine.fasano@unina.it (G.F.); domenico.accardo@unina.it (D.A.)

**Keywords:** unmanned aerial vehicles, visual detection, visual tracking, template matching, morphological filtering, cooperative UAV applications, autonomous navigation

## Abstract

This paper presents a visual-based approach that allows an Unmanned Aerial Vehicle (UAV) to detect and track a cooperative flying vehicle autonomously using a monocular camera. The algorithms are based on template matching and morphological filtering, thus being able to operate within a wide range of relative distances (i.e., from a few meters up to several tens of meters), while ensuring robustness against variations of illumination conditions, target scale and background. Furthermore, the image processing chain takes full advantage of navigation hints (i.e., relative positioning and own-ship attitude estimates) to improve the computational efficiency and optimize the trade-off between correct detections, false alarms and missed detections. Clearly, the required exchange of information is enabled by the cooperative nature of the formation through a reliable inter-vehicle data-link. Performance assessment is carried out by exploiting flight data collected during an ad hoc experimental campaign. The proposed approach is a key building block of cooperative architectures designed to improve UAV navigation performance either under nominal GNSS coverage or in GNSS-challenging environments.

## 1. Introduction

Machine vision systems and algorithms represent an essential tool for several applications involving the use of Unmanned Aerial Vehicles (UAVs) [1,2]. On one side, these techniques are frequently used to recover information about the surrounding scene, meaning that visual cameras are payloads required to accomplish the mission goal. This is the case of several civilian/military applications like infrastructure monitoring [3], surveillance of coastal areas [4] and 3D mapping [5]. On the other side, visual technologies and algorithms play a key role in improving UAV inner functionality, e.g., in terms of autonomous guidance, navigation and control and situational awareness. Indeed, autonomous localization [6,7], autonomous landing [8,9], obstacle detection [10,11] and sense and avoid [12,13] are just a few examples of UAV functionalities enabled or enhanced by vision-based systems. Besides standalone UAV operations, vision-based techniques play a crucial role also in activities carried out by UAV swarms. Specifically, they can be used to enable cooperative 3D mapping [14] and navigation aiding, either in GNSS-challenging/GNSS-denied environment [15,16] or under nominal GNSS coverage [17]. In this respect, the capability for a UAV to detect and track other cooperative aerial vehicles in a sequence of camera frames is a key factor. This task is investigated in this paper.

Detection and tracking of airborne objects from a UAV has recently become an important research topic in the open literature. Existing approaches can be mainly classified considering whether the target is an unknown flying object or a cooperative, known vehicle. The former case, for instance, is relevant to sense and avoid applications for which the necessity to maximize the detection range (thus ensuring adequate time to collision) places the focus on long-range scenarios. This means that the airborne target will cover very few pixels. For such an application, morphological filtering has proven to be a critical component of the architectures adopted for image processing [13,18,19]. Instead, concerning cooperative applications, the target will move through a wide range of distances from the camera, thus covering larger portions of its field of view. While the extended nature of the target gives the possibility to select a wider range of image processing tools, its cooperative nature can be exploited to enhance the target detectability, e.g., (i) by installing onboard a pattern of active Light Emitting Diodes (LEDs) [14] or passive artificial markers [20] or (ii) by relying on color-based information if the target has a distinctive, highly-recognizable color signature [21]. Clearly, the use of active LEDs comes at the expense of additional power and weight (which may not be compatible with the system requirements), while passive markers may be detectable only at very short distances (e.g., for relative localization in cooperative swarms’ applications [22]). On the other hand, the use of color-information-based methods is limited to targets characterized by a monotone, unique color, and their reliability may be sensitive to illumination changes. For these reasons, an innovative vision-based architecture for UAV-UAV detection and tracking is presented in this paper. The proposed architecture integrates different image processing concepts, i.e., template matching, morphological filtering and template updating, and it takes advantage of the cooperative nature of the two UAVs to provide a solution to the detection and tracking task, which is robust against variations in illumination conditions, target scale and local background. Specifically, the cooperation is exploited in terms of the exchange of navigation data from the target UAV to the one embarking the camera through a dedicated communication link, which allows aiding the image processing functions. As it is clarified in more detail in Section 3, this function is of interest to improve UAV navigation performance when operating either under nominal GNSS coverage or in GNSS-challenging environments. The former case is relevant to applications, such as accurate 3D mapping or precise pointing of payloads, where a “chief” UAV can exploit vision and GNSS information from “deputy” aircraft to generate very accurate attitude estimates. The latter scenario is relevant to applications, e.g., infrastructure monitoring, in which a “son” UAV has to fly autonomously in areas (such as natural or urban canyons) where the GNSS-based position fix is unavailable or unreliable (not enough satellites in view, bad dilution of precision, off-nominal errors in the pseudo-range measurements induced by multipath phenomena), exploiting “father” UAVs under nominal GNSS coverage. In both cases, as shown for instance in [17], cooperative navigation can be exploited with a minimum of two aircraft (one tracker and one target) and a unidirectional communication link. The concept can then be scaled for multiple UAVs, and in that case, a centralized networking architecture is needed where multiple target UAVs transmit their navigation data to the tracker UAV, and the algorithmic architecture presented in this paper must be run on board the tracker for each target. Overall, in such scenarios, the main constraints of the proposed approach are the need to (1) ensure pixel-level estimation accuracy of the target line-of-sight, (2) optimize the trade-off between missed detections and false alarms, keeping the latter at a minimum, and (3) operate in a wide range of distances when the target UAV can occupy either a few pixels or much larger regions of interest in the focal plane, being robust with respect to the possibility of abrupt changes in illumination and background conditions.

The remainder of the paper is organized as follows. Section 2 provides an overview of related works in the open literature with a focus on the image processing techniques. Furthermore, the originality and the advantages of the proposed approach are highlighted and motivated. Section 3 presents the practical context in which the visual detection and tracking algorithms are developed. Specifically, the integration of this processing block within two navigation architectures, tailored to mission scenarios entrusted to cooperative UAVs, is detailed. Section 4 describes each image processing step with a focus on the aiding strategies based on the available information about the absolute and relative navigation states. Finally, Section 5 includes the description of the setup used for experimental data collection, as well as the presentation and discussion of the results.

## 2. Related Work: Visual Detection and Tracking

With a focus on the image processing chain, the detection and tracking task investigated in this paper has critical challenges related to the fact that the relative motion between the camera, the target and the surrounding scene can cause a significant and high-dynamic variation of illumination conditions (e.g., the detector may be fully/partially saturated for a non-negligible time frame), background characteristics (e.g., color, reflectivity, homogeneity) and target appearance (e.g., scale and color). Clearly, the proposed vision-based algorithms must demonstrate adequate robustness toward these problems.

Existing approaches from the open literature can be mainly classified into two categories: (1) direct/feature-based techniques and (2) machine learning methods. The former category refers to those algorithms trying to identify a specific region-of-interest in the image (usually identified by a rectangular bounding box) by looking for the best match with a reference representation of the target (typically called a template). Hence, these techniques are typically referred to as Template Matching (TM). Direct approaches exploit the intensity of local gradient information at each pixel [23], while feature-based methods rely on visual features (e.g., Harris corners [24]) tracked through a sequence of frames, and distinguished using descriptors (e.g., SIFT [25] or SURF [26]). On the other side, machine learning algorithms are based on the training, either off-line or on-line, of a neural network to discriminate the selected target from the background. Consequently, their main drawback is the huge amount of data typically required to train the network. The most important category of machine learning methods is given by deep learning networks [27], among which it is worth mentioning the Deep Learning Tracker (DLT) [28], which exploits the idea of splitting the tracking task into a coarse estimation (based on an off-line training) and an on-line refinement. Both direct/feature-based and machine learning approaches may be characterized by hierarchical or cascade architectures conceived to accelerate the convergence of the detection and tracking processes, e.g., by relaxing the achievable accuracy at distinct levels of the hierarchy or adopting a sequence of search tests (cascade) to gradually reduce the number of potential candidates up to a unique detection declaration. Cascade architectures may foresee the use of more complex image processing techniques while moving forward in the detection process.

It is worth mentioning the main recent works dedicated to the problem of detecting and tracking a moving target from a UAV. A vision-based approach for micro-UAV detection and distance estimation was proposed in [29]. The method is feature-based, and it exploits a trained cascade classifier for target detection. The performance attained using three distinct types of features are evaluated and compared over data collected both indoors and outdoors. The method is demonstrated to be suitable for real-time implementation, but its applicability is limited to a very close range (up to 25 m). Another algorithm based on visual features (i.e., the FAST, Features from Accelerated Segment Test, points [30]), but designed for moving target tracking from a UAV is presented in [31]. The method has three main steps (i.e., global matching and local tracking, local geometric filter and local outlier factor), which allow a target model (built based on the features extracted at the first frame) to be tracked through a sequence of frames by determining robust feature matches. An approach for the removal of inaccurate matches (outliers) is also foreseen. Real-time performance is demonstrated by means of several outdoor tests (50 flights) considering targets characterized by extremely distinct size and shape, in relative motion with respect to the UAV onboard camera. The main drawback is the lack of an autonomous strategy to detect the target in the first frame, since the Region-Of-Interest (ROI) is selected manually. Furthermore, the algorithm is not designed to track targets in the case of extremely fast relative dynamics, which can occur if the target is a cooperative UAV. A full image processing architecture for multi-target detection and tracking can be found in [32]. This approach is based on the estimation of the background motion with respect to the camera. Then, potential targets are identified and classified in the background subtracted image. The tracking function is finally augmented thanks to a Kalman filter, which forces temporal consistency between consecutive detections. The method is designed to limit false alarms arising from the background motion estimation; however, it is sensitive to missed detections in the case of a complex, cluttered background. Another target detection method for images collected by a moving camera that aims at combining object appearance information and motion cues was given by [33]. This approach takes full advantage of the temporal information derived from a sequence of frames, thus providing an innovative motion compensation strategy, which is highly robust to changes in the appearance of both the object and background. The algorithm can search for the target at different spatial scales simultaneously, although this setting may have a negative impact on the computational burden.

Given this framework, an original approach for cooperative UAV detection and tracking using a monocular camera installed onboard another UAV is developed and tested in this paper. From now on, the UAV with the onboard camera is called the “tracker”, while the cooperative vehicle to be tracked is the “target”. The proposed algorithms exploit the combination of multiple processing steps relying on TM and morphological filtering. This choice allows dealing with the problem of significant changes in the background, which can occur from frame to frame due to the fast UAV-UAV dynamics. Specifically, the TM algorithm quickly recovers the target position in the image plane when the background is homogenous, while the morphological filter serves as an aiding technique to cope with background changes either in the intensity or in the clutter level. The possibility of losing track of the target due to variations in the scene is further limited by the implementation of an effective method to update online the template without incurring the typical phenomenon of template drifting [34]. Another key original contribution is the fact that the techniques composing the proposed image processing chain take advantage of navigation hints, i.e., the attitude of the tracker and the UAV-UAV relative position. Specifically, these data are used: (1) to reduce the computational effort (which is a critical requirement for real-time implementation) by significantly limiting the search area to be covered; (2) to extend the algorithm applicability to a wide range of relative distances (i.e., from a few meters up to several tens of meters); (3) to improve robustness against false target detections, which may occur if the target is searched over the entire image plane. Clearly, a reliable communication link must be foreseen so that the target is able to exchange information about its coarse absolute positioning to the tracker. An additional innovative point about the proposed approach is that the image processing chain involves strategies for autonomous failure detection, as well as to recover the target after it falls temporarily out of the Field Of View (FOV), which allow increasing the overall robustness. These strategies are tuned to optimize the trade-off between false alarms and missed detections according to the requirement of the cooperative UAV applications of interest. This concept is clarified in the next section.

To summarize the overview of this section, the key features of the mentioned recent works dedicated to the problem of detecting and tracking a moving target from a UAV are collected in Table 1, including the approach proposed in this paper. The symbol N/A stands for not applicable.

## 3. Cooperative Multi-UAV Applications

Vision-based detection and tracking is a critical enabling technology for practical mission scenarios relying on swarms of cooperative UAVs. In fact, multi-UAV missions can exploit various relative sensing systems, such as Radio-Frequency (RF)-based ranging. In this framework, the main advantages relevant to vision systems are: (1) no additional sensors are needed on board the target UAV; (2) visual cameras are very small, light and inexpensive with respect to latest generation cm-level RF ranging systems; (3) they provide accurate line-of-sight information, which may be required in specific cooperative navigation applications.

The approach proposed in this paper is conceived to be integrated within two navigation architectures capable of enabling:

(1) high-accuracy attitude estimation for UAVs flying under nominal GNSS coverage [17];
(2) safe autonomous navigation for UAVs flying in GNSS-challenging environments [35].

**Figure 1:**
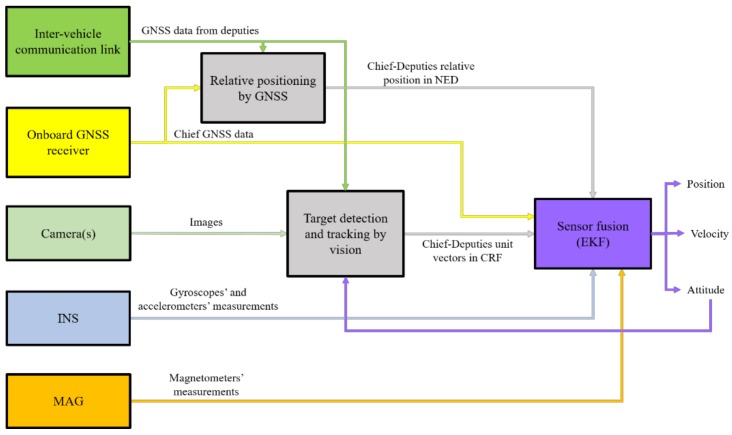
Block diagram of the navigation architecture for high-accuracy attitude estimation for UAVs flying under nominal GNSS coverage. Relative positioning is achieved by code/carrier-based differential GNSS.

In [17], the authors presented the concept of improving the absolute navigation performance of a UAV (chief) by exploiting a formation of cooperative flying vehicles (one/more deputies). Specifically, the proposed navigation architecture combines differential GNSS and relative sensing by vision within an original sensor fusion scheme based on an Extended Kalman Filter (EKF). Numerical simulations and experimental flight tests demonstrated the possibility of obtaining a heading uncertainty of about 0.1°, which is much better than the one achievable by integrating low-cost IMUs, GNSS and magnetometers. A block diagram of the full navigation architecture is given in Figure 1, where the chief is the tracker UAV, while the deputies are the targets of the visual detection and tracking block. The availability of Line-Of-Sight (LOS) information in both the inertial North-East-Down (NED) and Camera Reference Frames (CRF), provided by differential GNSS and image processing, respectively, allows significant improvement of the accuracy of the attitude estimation for the chief.

**Figure 2:**
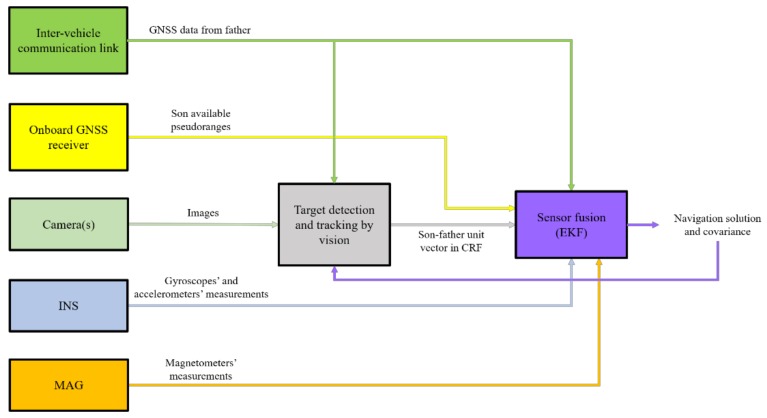
Block diagram of the architecture for safe autonomous navigation for UAVs flying in GNSS-challenging environments.

In [35], the authors presented the concept of enabling safe autonomous navigation of a UAV (son) flying in GNSS-challenging environments (e.g., natural/urban canyons where the GNSS position fix may not be enabled, or reliable, due to the lack of enough measurement) by exploiting cooperation with another UAV (father) flying under nominal GNSS coverage. Specifically, the absolute position of the father (provided through an inter-vehicle data-link), the son-father LOS (provided by the target visual detection and tracking block) and additional pseudo-ranges (if available at the GNSS receiver of the son) are combined within an original sensor fusion scheme based on an Extended Kalman Filter (EKF). Numerical simulations and experimental flight tests demonstrated the possibility of limiting the drift in the navigation solution (which would occur integrating only inertial measurements in absence of GNSS data), thus obtaining a performance level comparable with standard, standalone GNSS/INS approaches (even with two or three pseudo-range measurements). A block diagram of the full navigation architecture is given in Figure 2.

As anticipated in Section 2, the two block diagrams highlight how the visual detection and tracking function (which is the focus of this paper) benefits from navigation hints in both the considered architectures in order to improve computational efficiency and robustness. This aspect is further clarified in Section 4. Within the considered cooperative applications, the main requirement for the visual detection and tracking block is the necessity to optimize the trade-off between false alarms and missed detections. False alarms need to be kept at a minimum (nullified if possible) since they cause a significant loss of the navigation accuracy. On the other hand, a few missed detections may be acceptable if they do not compromise the integrity of the cooperative solution. Another key factor to consider is the need to operate in a wide range of distances when the target UAV can occupy either a few pixels or much larger ROIs in the focal plane. In terms of background and illumination, outdoor flight and low altitude operation lead to the possibility of abrupt changes in operating conditions. In all cases, LOS estimation accuracy is requested to be pixel-level, to fully exploit the potential of camera angular resolution.

## 4. Image Processing Algorithms

**Figure 3:**
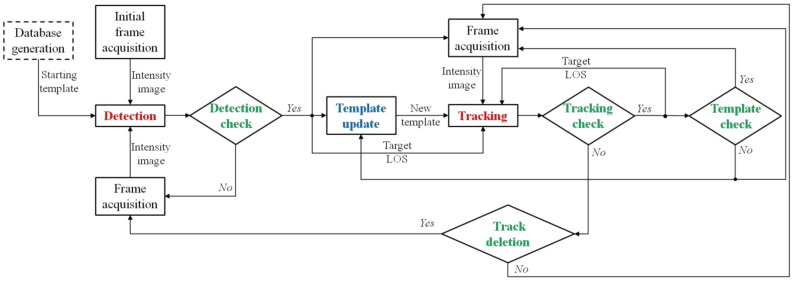
Flow diagram of the proposed approach for vision-based detection and tracking. The dashed, rectangular box indicates the off-line operation. Even if they are not reported in the chart, to avoid confusion, navigation data are provided as input to the detection and tracking blocks.

The flow diagram in Figure 3 describes in detail the visual detection and tracking block designed for the navigation architectures presented in Section 3. Besides the frame acquisition instructions, the proposed approach includes:

- an off-line “database generation” step enclosed in a dashed, rectangular box;
- two main processing steps, i.e., detection and tracking, highlighted in red;
- a supplementary processing step, i.e., template update, highlighted in blue;
- four decision points highlighted in green.

Both detection and tracking rely on the TM concept, which is the problem of searching for an image ROI that provides the best correlation score when matched to a template. The “starting template” is extracted from a database built off-line, which may be composed of either synthetic (generated by a camera simulator software) or experimental images. Clearly, the templates composing this database must be selected to adequately sample the range of relative distances of interest (which depends on the cooperative scenario) and, if possible, considering various conditions in terms of background and environmental illumination. The search in the database is used only by the detection algorithm. Instead, the tracking function exploits new templates generated on-line from the images under processing. The operation of generating the new template is entrusted to the “Template update” function, which is run each time a new detection is provided, as well as during tracking. Finally, the four decision points allow autonomously declaring success of the detection and tracking processes (“Detection check” and “Tracking check”, respectively), autonomously identifying loss of target position in the case of multiple, consecutive failures of the tracking step (“Track deletion”) and autonomously realizing the need to update the template to be searched in the subsequent frames during the tracking process (this necessity may be caused by a significant variation in the target and background appearance).

Details about the detection, tracking and template update steps, as well as about the conditions to be verified at each decision point are provided in the following sections. In this respect, it is worth outlining that the upcoming discussion will also clarify how the navigation hints available thanks to the cooperative nature of the proposed visual detection and tracking framework are used by each processing step to improve computational efficiency and robustness.

### 4.1. Detection

**Figure 4:**
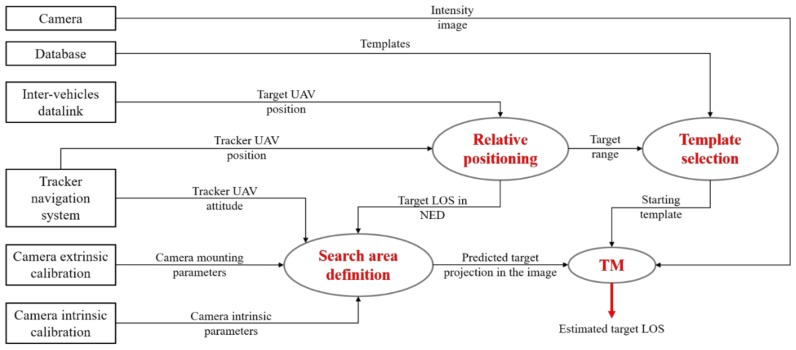
Flow diagram of the TM-based detection algorithm. The red arrow identifies the output of the process.

The algorithm in charge of the initial estimation of the LOS of the cooperative target UAV is based on the TM concept. In this respect, Figure 4 gives a detailed overview of the detection process.

Specifically, the TM function requires three inputs:

(1) an intensity (grey-level) image (*I*) acquired by the camera onboard the tracker UAV, whose horizontal and vertical size is indicated by *N_u_* and *N_v_*, respectively;
(2) a predicted estimate of the target projection in the image plane (*u_pr_*, *v_pr_*), which allows defining a limited area where the TM must be applied;
(3) a template (*T*), which is an intensity image with the size of the region of interest, purposely extracted from the database.

**Figure 5:**
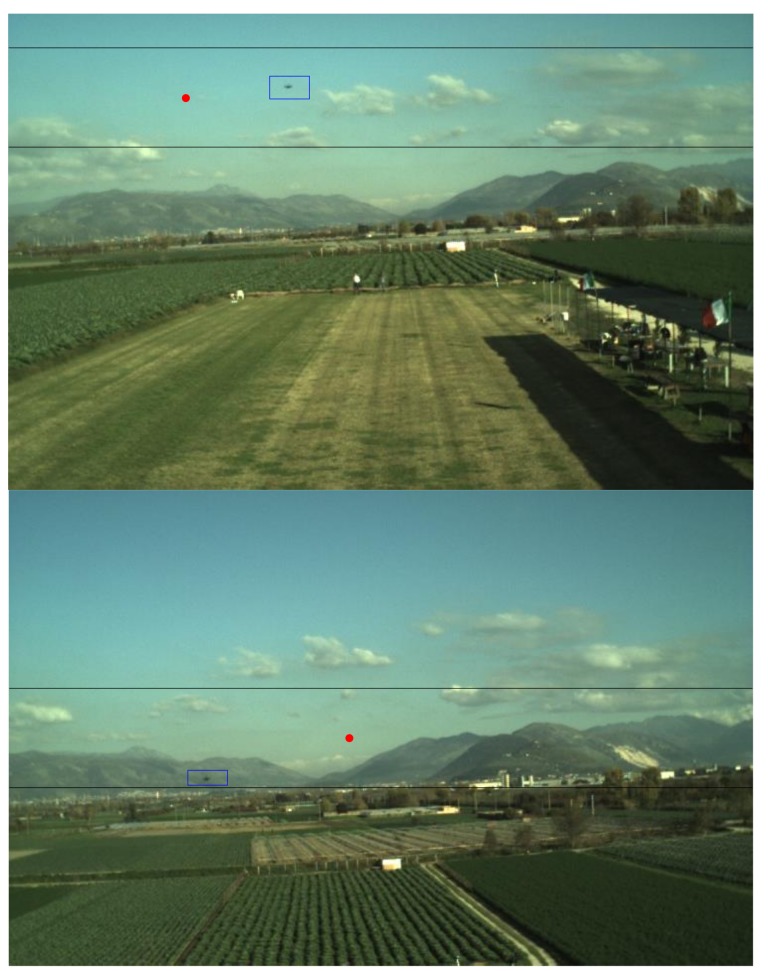
Examples of Template Matching (TM) search area for detection (image stripe delimited by two black lines). The target is enclosed in a blue box. The predicted position is highlighted by a red dot. *W_v,det_* is set to 100 pixel.

The role of the navigation hints, i.e., the set of data regarding the absolute state of the two vehicles suitable for template selection and definition of the TM search area, is now clarified. First, the absolute position of the vehicles is used by the relative position block to determine the target range (*ρ*) and the target LOS in NED (*ρ_n_*). The strategies that can be conceived and implemented within this block are determined by the type of data that the target vehicle is able to exchange with the tracker. With reference to the first navigation architecture discussed in Section 3 (see Figure 1), the two UAVs fly under nominal GNSS coverage. Consequently, *ρ_n_* can be estimated as the difference between the two GNSS position fix or exploiting Differential GPS (DGPS) and Carrier-Phase DGPS (CDGPS) algorithms. On the other hand, with reference to the second navigation architecture discussed in Section 3 (see Figure 2), the GNSS position fix is not available for the target vehicle. However, the absolute position determined by the onboard navigation filter can still be transmitted to the tracker, thus enabling the estimation of *ρ_n_* and *ρ*. Hence, the value of *ρ* is used to have an estimate of the size of the target in the image, thus allowing the selection of the starting template from the database (which is composed of target images corresponding to different values of relative distance, extracted from a dataset collected during previous, ad hoc flight tests). Instead, *ρ_n_* is exploited to obtain a prediction of the target LOS in the CRF (*ρ_c_*) using Equation (1),

(1) $${\underline{\rho }}_{c}={\underline{\underline{R}}}_{bc}{\underline{\underline{R}}}_{nb}{\underline{\rho }}_{n}$$

where *R_nb_* is the rotation matrix representing the attitude of the tracker UAV in NED (provided by its onboard navigation system), while *R_bc_* is the rotation matrix representing the attitude of the CRF with respect to the Body Reference Frame (BRF) (obtained by performing off-line the extrinsic calibration of the camera, thus determining its mounting parameters). At this point, the camera calibration matrix (*K*), obtained by performing (off-line) the intrinsic calibration procedure, can be used to estimate *u_pr_* and *v_pr_*, as shown by Equation (2),

(2) [uprvpr]=f(K__)[ρcuρczρcvρcz]

where *f* is a function of the camera calibration parameters [36], *ρ_cu_* and *ρ_cv_* are the cross-boresight components of *ρ_c_*, while *ρ_cz_* is the along-boresight component of *ρ_c_*. Once a prediction of the target position in the image is available, the TM search area for detection must be defined. To this aim, the uncertainty in the estimate of the tracker attitude used for prediction must be considered. Specifically, although *R_nb_* is typically expressed as a 321 sequence of Euler angles, heading, *ψ*, pitch, *θ*, and roll, *φ*, these three measurements do not have the same uncertainty. Indeed, the error in the heading estimate can be of several degrees mainly due to internal/external magnetic disturbances, and its value also depends on the experimented flight dynamics history. On the other hand, the roll and pitch uncertainties are typically bounded at the degree-level due to the accuracy in the gravity determination. Thus, the uncertainty in the value of *u_pr_* and *v_pr_* is limited to a few tens of pixels. This allows defining the TM search area as a stripe delimited by the interval (1, *N_u_*), horizontally, and (*v_pr_* − *W_v,det_*/2, *v_pr_* + *W_v,det_*/2), vertically. Some examples of search areas defined to perform detection are shown in Figure 5.

This strategy allows not only improving the time efficiency of the TM computation, but it also allows limiting the probability of false alarms. Moreover, the possibility of missed detection, if the target is not included in the horizontal image stripe, can be kept very low by properly selecting the value of *W_v,det_*, as will be shown in Section 5 by exploiting the experimental data collected for the test campaign.

The well-known Normalized Cross-Correlation (NCC) function (*γ*) [37] is then used to evaluate the correlation score at each pixel (*u*, *v*) in the TM search area as shown by Equation (3)

(3) $$\gamma (u,v)=\frac{\sum_{\begin{array}{l} x=1,\\ y=v_{pr}-W_{v,\det}/2 \end{array}}^{\begin{array}{l} N_{u},\\ v_{pr}+W_{v,\det}/2 \end{array}}\left[\left(I(x,y)-I^{*}{}_{uv}\right)\left(T(x-u,y-v)-T^{*}\right)\right]}{\sqrt{\sum_{\begin{array}{l} x=1,\\ y=v_{pr}-W_{v,\det}/2 \end{array}}^{\begin{array}{l} N_{u},\\ v_{pr}+W_{v,\det}/2 \end{array}}{\left(I(x,y)-I^{*}{}_{uv}\right)}^{2}\sum_{\begin{array}{l} x=1,\\ y=v_{pr}-W_{v,\det} \end{array}}^{\begin{array}{l} N_{u},\\ v_{pr}+W_{v,\det} \end{array}}{\left(T(x-u,y-v)-T^{*}\right)}^{2}}}$$

where I^*^_uv_ and T^*^ are the mean intensity values of the image within the stripe and of the template, respectively. Finally, the estimated target LOS can be obtained inverting Equation (2), using as input the point in the image plane where *γ* is maximum (*γ_max_*), identified by the pixel coordinates (*u_det_*, *v_det_*).

At this point, the detection process is declared successful if *γ_max_* is higher than a detection threshold (*τ_det_*). This check for the quality of the solution is entrusted to the decision point “Detection check” (see Figure 1). In this respect, the selection of the value of *τ_det_* is the result of a trade-off between missed detections and false alarms. A sensitivity analysis to assess the effect of *τ_det_* on detection is carried out in Section 5 using the available flight-testing data. Clearly, if the acceptance condition is not satisfied, the detection process shall be re-started by a new camera acquisition.

### 4.2. Tracking

**Figure 6:**
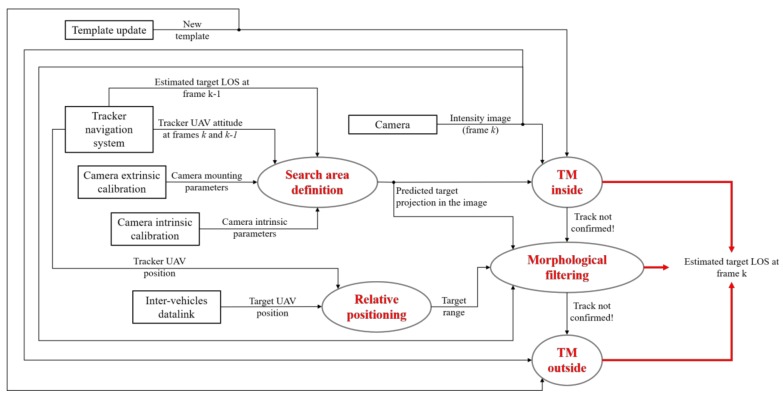
Flow diagram of the tracking algorithm based on TM and morphological filtering. The red arrows identify the output of the process.

Once detection is declared successful and a new template is extracted from the image (see Section 4.3 for a description of the template update function), the tracking process can be started. The goal of this step is to provide an estimate of the target LOS at the current frame (*k*), given the last available one corresponding to a previous frame (*k* − 1). In this respect, Figure 6 gives a detailed overview of the tracking process.

Differently from detection, tracking is entrusted to three distinct functions, namely TM-inside, morphological filtering and TM-outside, which are activated sequentially until a track is confirmed according to purposely defined failure detection strategies (managed within the decision point “Tracking check” in Figure 3). Clearly, if the track is not confirmed even after the third function is implemented, a new image is acquired, and the tracking process is restarted. An additional safety strategy is foreseen to cope with possible time lapses during which the target falls outside the camera FOV. This condition is verified by the decision point “Track deletion” in Figure 3. Specifically, if the track is not confirmed for a sequence of *N_d_* frames (the number depends on the frame rate), the track is deleted, and the detection process is restarted.

The three main functions of the tracking process are now described clarifying the role of the navigation hints.

#### 4.2.1. TM-Inside Processing

The TM algorithm is applied within a rectangular window in the image plane centered at a pixel (*u_pr_*, *v_pr_*) that represents the predicted position of the target. This prediction is obtained exploiting the target LOS estimated at the previous frame (*ρ_c_^k^*^−1^), as well as the variation of the attitude of the tracker UAV between *k* and *k* − 1. Indeed, the corresponding rotation matrixes (i.e., $\underline{\underline{R}}$ *_nb_^k^*^−1^ and $\underline{\underline{R}}$ *_nb_^k^*), provided by the onboard navigation system of the tracker, can be used to predict the target LOS at the current frame (*ρ_c_^k^*) using Equation (4).

(4) $${\underline{\rho }}_{c}{}^{k}={\underline{\underline{R}}}_{bc}{\underline{\underline{R}}}_{nb}{}^{k}\left({\underline{\underline{R}}}_{bn}{}^{k-1}{\underline{\underline{R}}}_{cb}{\underline{\rho }}_{c}{}^{k-1}\right)$$

**Figure 7:**
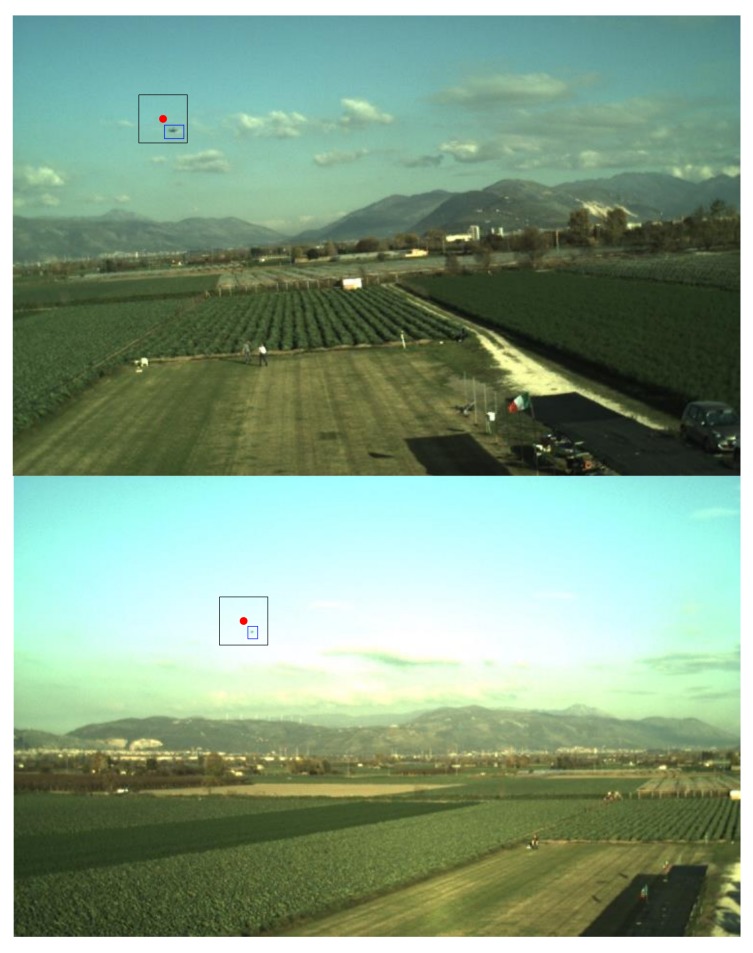
Examples of the TM search area for tracking (rectangular black window). The target is enclosed in a blue box. The predicted position is highlighted by a red dot. *W_u,tr_ and W_v,tr_* are both set to 50 pixels.

This equation assumes that the variation of the relative unit vector in NED between two successive frames is negligible. While this assumption is certainly applicable for smooth relative dynamics and if the frame rate is adequately high, the approach can be extended to the case of aggressive relative dynamics by using the velocities of the two aircraft estimated by the corresponding onboard navigation systems. Given *ρ_c_^k^*, the target position on the image plane can be predicted using Equation (2). Since the attitude variation among consecutive frames is much more accurate than the absolute attitude information provided by inertial navigation systems, the search area for the TM-inside function can be defined as a rectangular box centered at (*u_pr_*, *v_pr_*), with a horizontal and vertical size equal to *W_u,tr_* and *W_v,tr_*, respectively. Some examples of search area definition for tracking are shown in Figure 7.

At this point, the NCC function for tracking (*γ_tr_*) can be computed using Equation (5).

(5) $$\gamma_{tr}(u,v)=\frac{\sum_{\begin{array}{l} x=u_{pr}-W_{v,\mathrm{tr}}/2,\\ y=v_{pr}-W_{v,\mathrm{tr}}/2 \end{array}}^{\begin{array}{l} u_{pr}+W_{v,\mathrm{tr}}/2,\\ v_{pr}+W_{v,\mathrm{tr}}/2 \end{array}}\left[\left(I(x,y)-I^{*}{}_{uv}\right)\left(T(x-u,y-v)-T^{*}\right)\right]}{\sqrt{\sum_{\begin{array}{l} x=u_{pr}-W_{v,\mathrm{tr}}/2,\\ y=v_{pr}-W_{v,\mathrm{tr}}/2 \end{array}}^{\begin{array}{l} u_{pr}+W_{v,\mathrm{tr}}/2,\\ v_{pr}+W_{v,\mathrm{tr}}/2 \end{array}}{\left(I(x,y)-I^{*}{}_{uv}\right)}^{2}\sum_{\begin{array}{l} x=u_{pr}-W_{v,\mathrm{tr}}/2,\\ y=v_{pr}-W_{v,\mathrm{tr}}/2 \end{array}}^{\begin{array}{l} u_{pr}+W_{v,\mathrm{tr}}/2,\\ v_{pr}+W_{v,\mathrm{tr}}/2 \end{array}}{\left(T(x-u,y-v)-T^{*}\right)}^{2}}}$$

As in the case of detection, the estimated target LOS can be obtained inverting Equation (2), using as input the point in the image plane where *γ_tr_* is maximum (*γ*_max_), identified by the pixel coordinates (*u_tr_*, *v_tr_*). The reliability of the solution provided by the TM-inside function is verified within the dedicated decision point (“Tracking check” in Figure 3). Specifically, the track is confirmed if *γ*_max_ is higher than a tracking threshold (*τ_tr,in_*). If the check is positive, the possibility to update the template is investigated (see Section 4.3). Otherwise, the tracking process continues with the morphological filter.

#### 4.2.2. Morphological Filtering

Even if the target is enclosed in the search area used to apply the TM-inside function, the target declaration may be missed due to variation in the background and/or environmental illumination. For this reason, if *γ*_max_ < *τ_tr,in_*, an algorithm based on morphological filtering is applied to carry out an additional search in the previously-defined area. Before entering the details of this algorithm, it is worth outlining that the morphological filter can be either bottom-hat or top-hat depending on whether the target is darker or brighter than the local background. In this work, the frame of the target UAV used for experimental validation of the proposed approach (see Section 5) is mostly black (besides two arms, which are dark metalized blue). Consequently, the target is always darker than the local background, thus allowing the use of a bottom-hat filter only. In the most general case, the target may have a completely different brightness depending on the direction of observation and illumination conditions or due to shadowing effects. In such a case, a different morphological filtering operator, e.g., a combination of the top-hat and bottom-hat filters (as done in [13]), shall be applied. With regards to the navigation hints, the size of the rectangular mask used by the morphological filtering operator (which is an all-ones matrix) is scaled based on the available range estimate (see Figure 6), thus being consistent with the number of pixels occupied by the target in the horizontal and vertical direction. Once the bottom-hat filter is applied, the resulting image window (*I_tr,BH_*) is binarized to remove the background. The grey-level threshold used for binarization (*τ_bin_*) is evaluated based on a statistical analysis of *I_tr,BH_* as shown by Equation (6),

(6) $$\tau_{bin}=\mu_{BH}+n_{BH}\sigma_{BH}$$

where *μ_BH_* and σ*_BH_* are the mean and standard deviation of *I_tr,BH_*, respectively, while *n_BH_* is a tuning parameter. Specifically, the larger the value of *n_BH_* is, the darker the foreground (i.e., the target) must be to allow extraction. In this work, a large value of *n_BH_* is convenient (e.g., 6 ÷ 8), the target being a particularly dark object. This choice also allows minimizing the risk of false alarms. Hence, the binarized image window is further processed using the distance transform and labeling operators to merge connected regions (based on the eight-pixel connectivity) and separate distinct candidates. Clearly, a minimum size is required for the extracted objects to be considered as reliable target candidates. At the end of this process:

(a) no image regions are extracted → the target is declared to be out of the search window;
(b) a single image region is extracted → the centroid is assigned as the estimated target position in the image (*u_tr_*, *v_tr_*);
(c) multiple image regions are extracted → the algorithm is not able to solve the ambiguity between the target and other objects (outliers).

**Figure 8:**
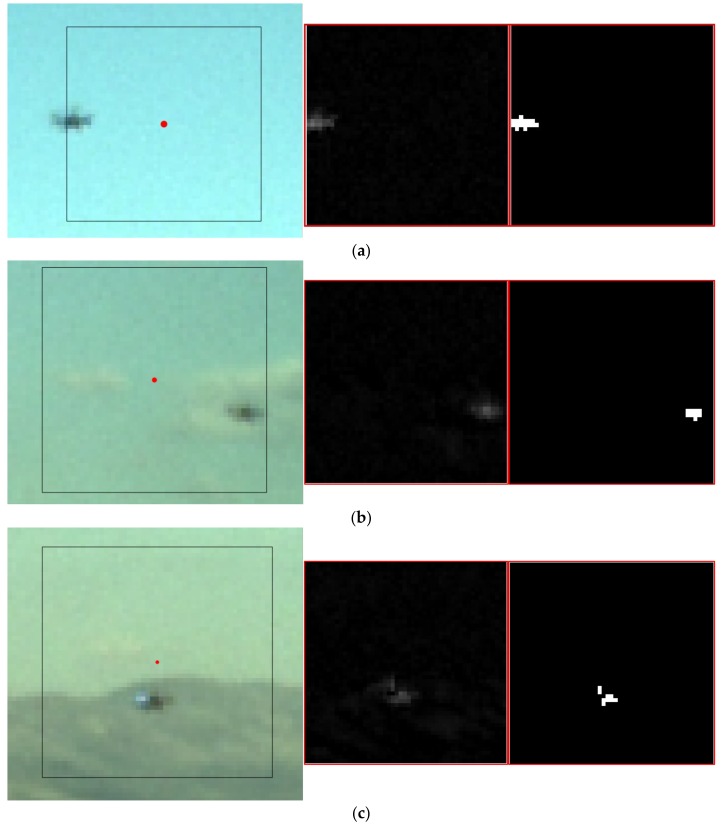
Examples of applications of the morphological filtering function within the tracking process. (Left) Zoom of the acquired color image. The prediction (red dot) and the search area (black box) are highlighted. (Center) Search window after applying the bottom-hat filter. (Right) Binarized search window. (**a**) The TM fails since the target is partially outside the box, thus causing a reduction in the Normalized Cross-Correlation (NCC) score. (**b**) The TM fails since the target is partially hindered by a cloud, thus causing again a reduction in the NCC score. (**c**) The TM fails due to the sudden change in the background (from the sky to the mountain). The smaller extracted region (on the left) is automatically discarded by the algorithm as an outlier due to its limited size.

Both in Cases (a) and (c), the target position in the image plane is not determined, and the next step of the tracking process must be activated. In conclusion, it is worth observing that, while the morphological filtering operator is particularly effective within a limited image region, it is not applied to the entire image since it can easily lead to false alarms. Figure 8 shows examples of frames where the morphological filter can extract the target despite the failure of the TM-inside function.

#### 4.2.3. TM-Outside Processing

If the target location on the image plane is not declared after the first two steps of the tracking process, an additional search is carried out applying the TM on the entire image. The target LOS is estimated from the pixel (*u_tr_*, *v_tr_*) where *γ_tr_* is maximum (*γ*_max_), and the track is confirmed if *γ*_max_ is larger than a threshold (*τ_tr,out_*). Clearly, this solution can increase the risk of false alarms. This risk can be mitigated, for instance, by selecting a higher value for *τ_tr,out_* than *τ_tr,in_*. On the other hand, it is worth noticing that is very difficult for the target to fall outside the search area if the camera frame rate is high enough to cope with the relative dynamics between the two flying vehicles.

### 4.3. Template Update

Once a track is confirmed by the TM-inside function, before skipping to the next frame, the proposed approach foresees the possibility to further update the template so that it can be better adapted to frame-by-frame variation of the background (adaptive TM). Specifically, the template is updated if the peak value of *γ_tr_* (*γ*_max_) satisfies the criterion shown by Equation (7):

(7) $$\tau_{tr,in}<\gamma_{\max}<\tau_{upd}$$

where *τ_upd_* is a new threshold introduced for template updating. This condition derives from the fact that if *γ*_max_ is particularly high (e.g., >0.9), it means that the target appearance in the image plane has not changed, and consequently, the current template can be exploited also in the next frame. If the condition stated by Equation (7) is satisfied, first, a rectangular image region, centered at (*u_tr_*, *v_tr_*) and having the same size as the current template is extracted. Then, to avoid the typical phenomenon of template drifting (i.e., the target gradually moves outside of the template), the center of the target shall be determined and correctly re-positioned in the image region representing the template. This is done using the Harris corner detector and extracting the highest quality corner.

## 5. Flight Test Campaign

This section aims at presenting the experimental performance assessment attained by field-testing the approach for visual detection and tracking described in the previous section. This is done exploiting experimental flight data collected using a couple of rotorcraft UAVs.

### 5.1. Experimental Setup

**Figure 9:**
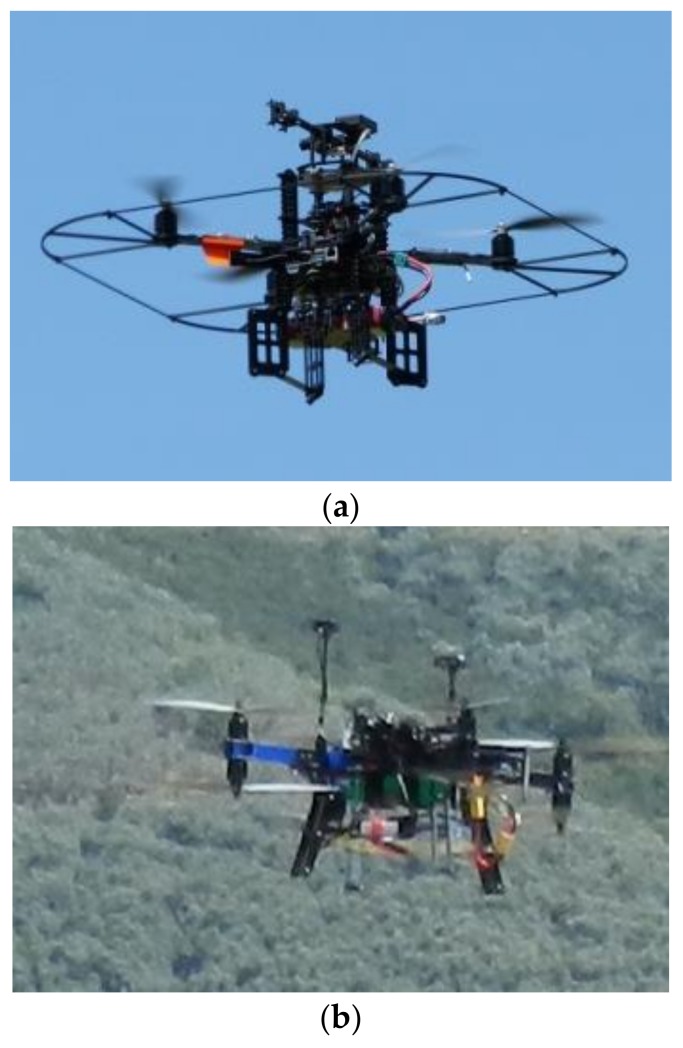
(**a**) AscTec Pelican^TM^ quadcopter used as tracker UAV. (**b**) 3DR X8+^TM^ octocopter used as target UAV.

The tracker UAV is a customized version of the Pelican^TM^ quadrotor (Figure 9a) from Ascending Technologies^TM^, which is equipped with an autopilot, an onboard computer (AscTec Mastermind^TM^), a GPS receiver and a set of low-cost MEMS sensors. The customization is given by the installation of an additional GPS single frequency receiver (uBlox LEA-6T^TM^) with raw measurements capabilities, an auxiliary GPS antenna and a 752 × 480 miniaturized CMOS forward looking camera (Matrix vision BlueFox^TM^ MLC200wC). This latter sensor is used for detection and tracking purposes. The additional GPS receiver and the camera have been connected to the Mastermind computer via a USB link. The target UAV is a customized version of the 3DR X8+^TM^ octocopter (Figure 9b), also equipped with the same auxiliary GPS system as the Pelican. Furthermore, an Odroid XU4^TM^ embedded CPU is installed onboard for data processing and storage. The adopted test strategy is based on the concept of data acquisition for off-line processing. Thus, no real-time data link among the UAVs is needed, and proper acquisition software has been developed to save all the data with an accurate time-tag based on the CPU clock. This time-tag is also associated with GPS measurements (including GPS time) gathered with very small latency, which enables accurate synchronization of all data acquired on each flying platform.

It is worth outlining that a V2V communication link shall be foreseen for the cooperative UAV applications where the proposed architecture must be run in real time. However, communication requirements are not particularly demanding due to the extremely limited amount of data (up to a few Kbits per second) that needs to be transmitted to exchange in line-of-sight basic navigation parameters. Thus, technologies available on board small UAVs can be exploited.

### 5.2. Flight Tests Description

**Figure 10:**
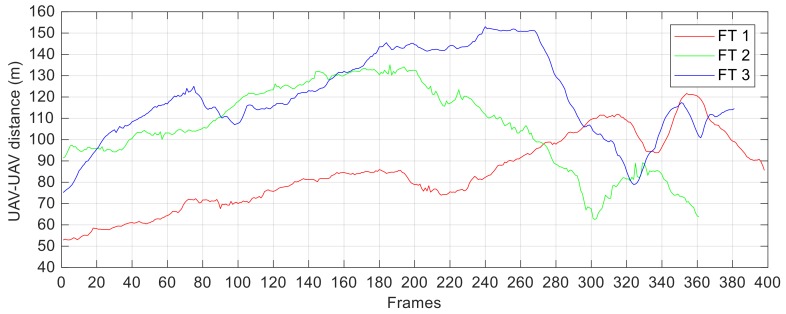
Relative distance computed by the relative positioning block during the three conducted Flight Tests (FTs).

The experimental campaign under analysis has been used to demonstrate the potential of the cooperative navigation architecture for applications under nominal GNSS coverage (see Figure 1) re-called in Section 2. Consequently, the two UAVs are flown to obtain a relative distance varying in a wide range of the order of several tens of meters during the three Flight Tests (FTs). Indeed, such baseline values allow the navigation architecture to satisfy very strict requirements in terms of the attained heading accuracy (i.e., pointing of the tracker UAV) [17]. The target range (*ρ*), used by the visual detection and tracking algorithms as the navigation hint, is computed by the relative positioning block as the difference between the GNSS position fix available at the two vehicles. The result of this operation is shown in Figure 10.

Overall, *ρ* ranges from a minimum of 53 m (the target occupies a rectangular ROI of 15 × 10 pixels) to a maximum of 153 m (the target occupies very few pixels). This allows testing the proposed visual architecture while coping with significant variations in the target scale. Another factor determining challenging conditions in terms of target appearance is the environmental illumination, which changes continuously during each flight (due to variations in the camera/Sun relative geometry and camera shutter time caused by the tracker UAV dynamics), as well as from flight to flight. Moreover, during each FT, a series of 360° turns is commanded to the tracker UAV, thus being able to assess the capability of the proposed architecture:

- to recognize periods of target absence from the FOV autonomously;
- to recover the target autonomously by re-starting the detection process.

The visual camera on board the Pelican is operated with a frame update rate of 1 Hz (since image acquisition is triggered by the GPS receiver to have synchronous data). This aspect, coupled with a particularly aggressive relative dynamics during each FT, allows demonstrating the robustness of the strategies, based on the use of navigation hints, conceived and implemented to aid the purely visual algorithms. A summary of the main information regarding the three FTs is given by Table 2, where *N* is the total number of analyzed frames, while *N_IN_* and *N_OUT_* represent the instances where the target is inside and outside the FOV, respectively. In this respect, it is worth outlining that FT 2 and FT 3 provide more challenging conditions than FT 1, especially due to the larger baselines. The entity of the target average displacement (Δ*u,* Δ*v*) between subsequent frames, which the tracking algorithm must cope with, is also highlighted.

**Figure 11:**
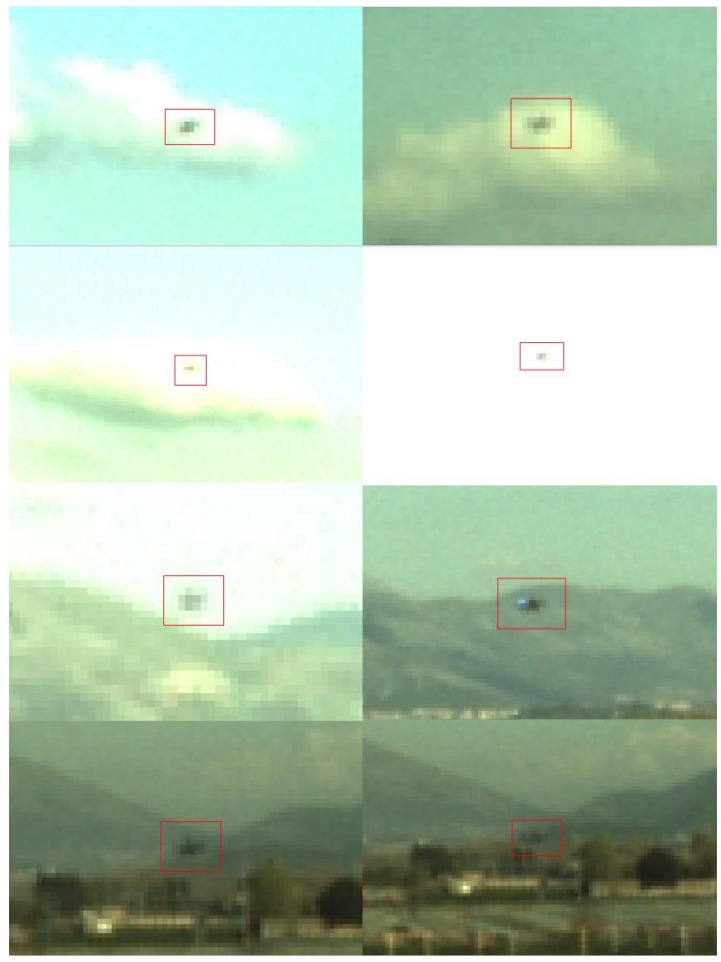
Examples of acquired frames where illumination or local background conditions are challenging for detection and tracking. Each sub-image is obtained zooming the corresponding frame around the target (which is highlighted by a red, rectangular box).

Finally, Figure 11 is a collection of frames from the three FTs. Each sub-image is zoomed around the target to highlight the wide variability of the local background (e.g., sky, clouds and vegetation), as well as some particularly challenging conditions (e.g., target partially hindered by clouds, target within a saturated ROI in the image plane) with which the proposed algorithms must deal. 

### 5.3. Performance Assessment: Detection Algorithm

**Figure 12:**
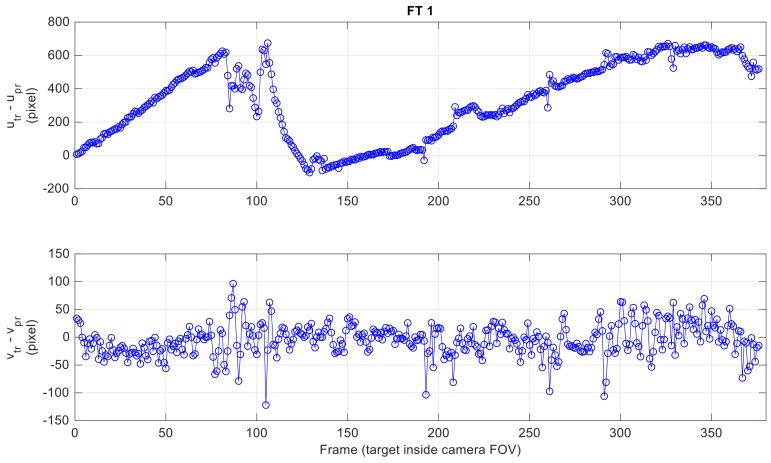
Horizontal (**up**) and vertical (**down**) error in predicted target position to carry out detection for FT 1.

First, it is interesting to demonstrate the applicability of the strategy adopted to define a reduced search area in the image (i.e., a horizontal stripe) exploiting the available hints from the navigation systems of the two vehicles. To this aim, the error between the actual target position in the image plane (*u_tr_*, *v_tr_*) and the prediction computed using Equations (1) and (2) is evaluated for the three FTs. Clearly, only the frames where the target is enclosed in the camera FOV are considered. Figure 12 shows the estimated prediction error for each considered frame in FT 1, while a statistical summary of this error for all FTs is given by Table 3.

As anticipated in Section 4.1, due to the poor accuracy in the heading information provided by the onboard autopilot of the Pelican, the horizontal prediction is completely unreliable to aid the detection process. Instead, the TM search area can be vertically limited to a horizontal image stripe centered on *v_pr_*. For instance, if *W_v,det_* is set to 100 pixels, the probability that the target falls outside the horizontal image stripe is 9%, 23% and 3% for the three FTs, respectively. The larger number for FT 2 is motivated by the more aggressive relative dynamics that characterizes the flight (as also shown in Table 3 by the large value of the standard deviation of the vertical prediction error, i.e., 67.1 pixels).

The detection performance is now analyzed for the three FTs. To this aim, four performance parameters are defined below.

- Percentage of Missed Detections (MD), computed as the ratio between the number of frames in which the target is wrongly declared to be outside the image plane (i.e., it is not detected even if it is present in the image) and the total number of analyzed frames.
- Percentage of Correct Detections (CD), computed as the ratio between the number of frames in which the target is correctly declared to be inside the image plane (the detection error is lower than a pixel threshold, *τ_pix_*, both horizontally and vertically) and the total number of analyzed frames.
- Percentage of False Alarms (FA), computed as the ratio between the number of frames in which the target is wrongly declared to be inside the image plane (i.e., it is detected even if it is not present in the image) and the total number of analyzed frames.
- Percentage of Wrong Detections (WD), computed as the ratio between the number of frames in which the target is correctly declared to be inside the image plane, but the detection error is larger than *τ_pix_* both horizontally and vertically, and the total number of analyzed frames.

**Figure 13:**
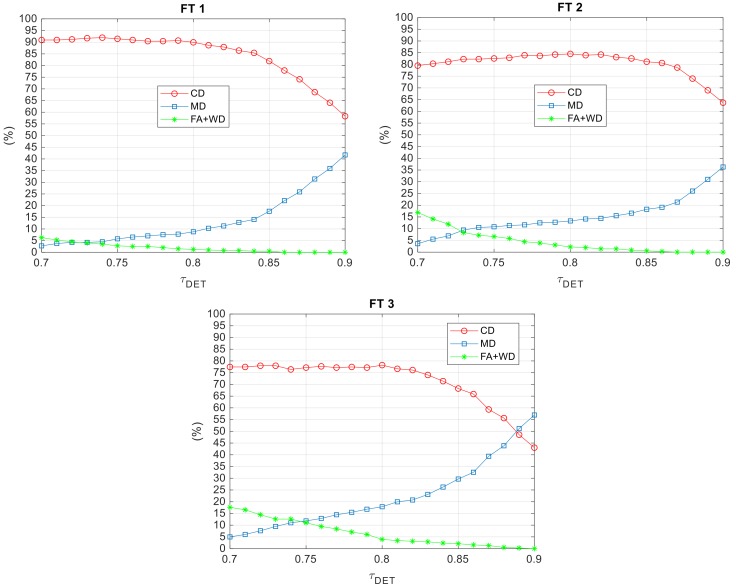
Performance parameters as a function of *τ_det_* for the three FTs. *W_v,det_* and *τ_pix_* are set to 100 and 10 pixels, respectively. CD, Correct Detections; MD, Missed Detections; FA, False Alarms; WD, Wrong Detections.

First, the effect on performance of selecting *τ_det_* is analyzed. Specifically, the behavior of the above-defined performance parameters while *τ_det_* varies from 0.7–0.9 is depicted by Figure 13. In this respect, while CD and MD are treated separately, the sum of FA and WD is taken as a whole. This choice is related to the fact that both FAs and WDs represent “severe” failures, i.e., they lead to inconsistent measurements of the target LOS, which could seriously compromise the operation of the navigation filter. On the other hand, MDs represent “more tolerable” failures provided that a CD is declared in one of the subsequent frames.

Overall, the selection of the most convenient value of *τ_det_* is determined by the need to (i) minimize FAs and WDs and (ii) optimize the trade-off between CDs and MDs. In this respect, results show that the maximum value of CD can be obtained setting *τ_det_* around 0.8 (90.0% FT 1, 84.5% FT 2, 78.2% FT 3). While this choice leads to a residual number of FAs and WDs, the occurrence of these “severe” failures can be pushed to zero increasing *τ_det_*. Of course, this comes at the expense of an increase in MD. As expected, a performance worsening can be noticed moving from FT 1 and FT 3. This is mainly caused by the larger UAV-UAV distances, but also by more frequent changes in the local background, characterizing the second and third flights. The latter aspect is important since the database is composed of target images selected from visual datasets collected during previous, ad hoc flight experiments. Specifically, a set of images with the target located at different ranges is extracted. During this database generation process, images with low target contrast with respect to the local background are discarded. This choice is motivated by the fact that it is not convenient to try enlarging the database with target images characterized by more complex background and illumination conditions. Indeed, such a solution would cause an increase in the computational complexity without necessarily improving detection performance. It is worth highlighting that although the proposed method for database generation may be restrictive if the target is a non-cooperative flying object, it is reasonable considering the cooperative nature of the UAV applications of interest to this work.

**Figure 14:**
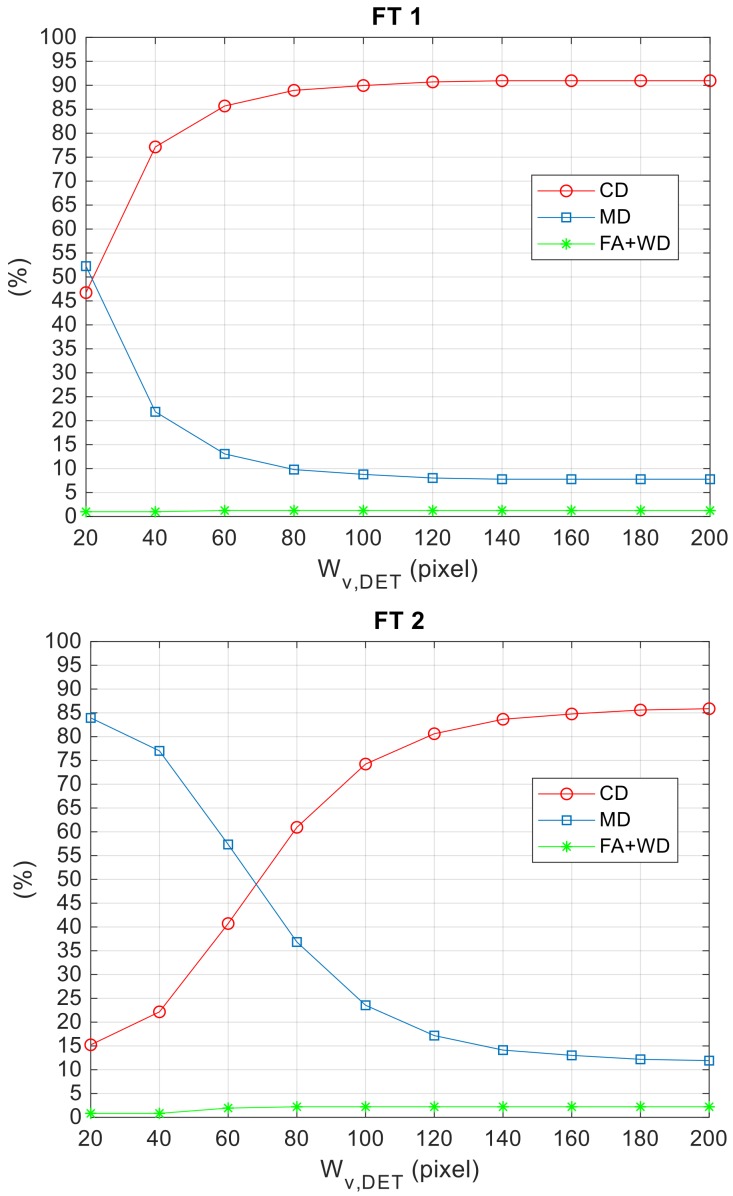
Performance parameters as a function of *τ_det_* for the three FTs. *W_v,det_* and *τ_pix_* are set to 100 and 10 pixels, respectively.

Finally, it is interesting to show the effect on detection performance of varying *W_v,det_*, i.e., the width of the image stripe selected as the search area for TM. Specifically, *W_v,det_* is varied between 20 and 200 pixels, and the results are collected in Figure 14 for FT 1 and FT 2.

The number of CDs (MDs) increases (reduces) asymptotically with *W_v,det_* due to the lower probability for the target to fall outside the TM search area. The lower value of *W_v,det_* needed for FT 1 to maximize the percentage of CDs with respect to FT 2 is justified by the more aggressive relative dynamics characterizing the second flight (see the statistics of the vertical prediction error in Table 3). Overall, it is possible to state that the adopted aiding strategy based on navigation hint allows limiting the computational burden without compromising detection performance, while selecting *W_v,det_* in a wide interval of values.

### 5.4. Performance Assessment: Tracking Algorithm

**Figure 15:**
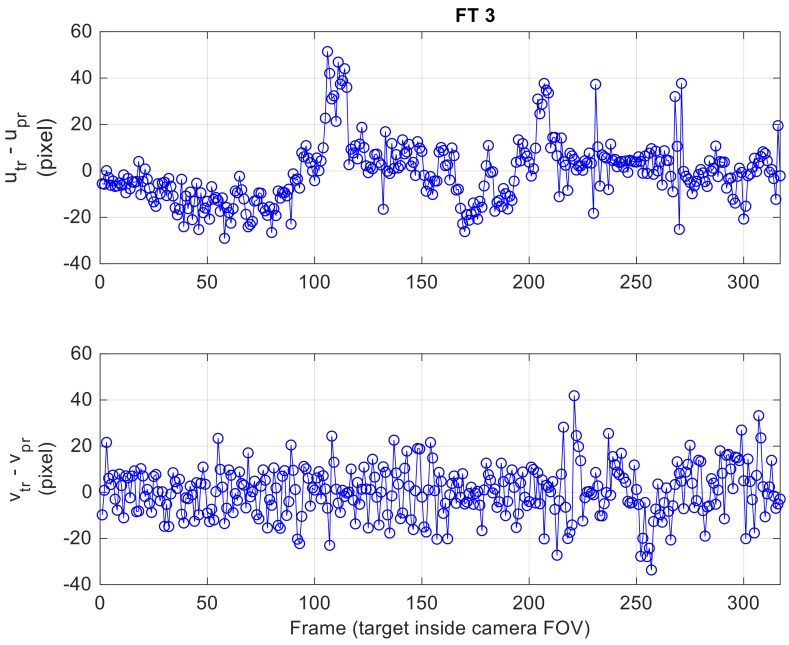
Horizontal (**top**) and vertical (**bottom**) error in predicted target position to carry out tracking for FT 3.

First, the applicability of the aiding strategy based on navigation hints, conceived to restrain the image area monitored by the TM-inside function and morphological filtering, is verified. In this case, the TM search is limited to a rectangular window of size (*W_u,tr_*, *W_v,tr_*) centered at the target position in the image plane (*u_pr_*, *v_pr_*) predicted using Equations (2) and (4) (which exploit the last available target LOS estimate and the attitude variation between the corresponding frame and the current one). Hence, it is interesting to analyze quantitatively the prediction error. To make this analysis independent of the performance of the visual detection and tracking architecture, the prediction is done considering the attitude measurements from the autopilot of the Pelican at each frame and the true target position at the previous frame. Figure 15 shows the prediction error for each frame where the target is enclosed in the FOV for FT 3, while a statistical summary of this error for all FTs is given by Table 4.

The results of this statistical analysis confirm that the aiding strategy based on navigation hints conceived for target tracking can be extremely effective. Indeed, if *W_u,tr_* and *W_v,tr_* are set to 50 pixels, the target prediction will fall in the TM search area (i.e., the prediction error is less than 25 pixels both horizontally and vertically) for 90% of the analyzed frames for each FT. This result is achieved despite considering a limited frame rate of 1 Hz. Clearly, the quality of the prediction can be improved operating at the much higher frame rates provided by state-of-the-art cameras (e.g., order of tens of fps). Finally, it is worth outlining that the validity of this analysis is not compromised by the choice of using the true target position instead of the estimated one at the previous frame since the estimation error in case of CDs is of the order of a few pixels (as is shown in the following part of this section).

At this point, the performance of the full visual detection and tracking architecture is analyzed for the three FTs (clearly, in the following, the last-available estimated target LOS is used to define the TM search area during the tracking phase). Again, the adopted performance parameters are the percentages of CD, MD, FA and WD, and *τ_pix_* (i.e., the threshold to declare CD or WD) is set to 10 pixels. With regards to the selection of the operational parameters of the detection and tracking algorithms, defined in Section 4, the following considerations can be made. Based on the performance of the detection algorithm shown in the previous section, *W_v,det_* is set to 100 pixels, while the same value of 0.86 is set for the three correlation thresholds. Although this latter choice does not allow optimizing the number of CDs, it is justified by the fact that a very large value of the correlation thresholds can lead very close to zero the occurrence of “severe” detection/tracking failures (i.e., FAs and WDs). Concerning the remaining parameters, *W_u,tr_* and *W_v,tr_* are set to 50 pixels, based on the data collected in Table 4. On the other hand, *τ_upd_* is assigned considering that it determines the number of frames at which the template is updated during the detection and tracking process (*N_upd_*). In this respect, if *τ_upd_* is lower than or equal to the correlation thresholds, the template is updated only at the first detection or if the detection function is recalled (according to the architecture in Figure 3). Instead, if *τ_upd_* gets larger than the correlation thresholds, *N_upd_* increases (i.e., updates can occur also during the tracking process). Hence, *τ_upd_* is set to 0.87, which determines a good trade-off between the need to update the template to deal with sudden changes in the local background, on one side, and the need to minimize the risk of selecting the wrong object as the template, e.g., in the case of FAs or WDs, on the other side. Hence, the results of the detection and tracking process are summarized in Table 5.

Although the best performance in terms of CDs is achieved for FT 1 (95.7%), which is not affected by either FA or WD, the number of “severe” failures is also very small for the remaining FTs, i.e., six and four frames for FT 2 and FT 3, respectively. This result is particularly promising considering that each of the three FTs lasts more than 6 min, thus containing more than 360 frames (see Table 2). The lower success rate achieved for FT 2 and FT 3 is justified by the more challenging conditions to deal with (especially in terms of target range), as highlighted in Section 5.1. Consequently, the template is updated more and more times (11 and 20, respectively) to limit FAs and WDs. If the attention is focused on FT 3, despite the very low number of FAs and WDs, the percentage of MDs may not be acceptable. To limit the occurrence of MDs, the correlation thresholds must be decreased, e.g., they can be set to 0.81, which is the largest value of *τ_det_* before the percentage of CD starts dropping down (see Figure 13). If *τ_upd_* is varied from 0.86–0.90, the performances of the detection and tracking process are as summarized in Table 6. The results show that if the correlation thresholds are lowered to reduce MDs, high values of *τ_upd_* are required to get satisfying performance steadily. For instance, if *τ_upd_* is 0.90, the template is updated 82 times. Compared to the results in Table 5 (last row), the increase in CDs (i.e., from 75%–85%) and the reduction of MDs (i.e., from 24%–12%) are paid in terms of more failures, i.e., ten (composed of eight FAs and two WDs) instead of four. Clearly, this risk shall be considered by the user when selecting the operational parameters of the algorithm.

**Figure 16:**
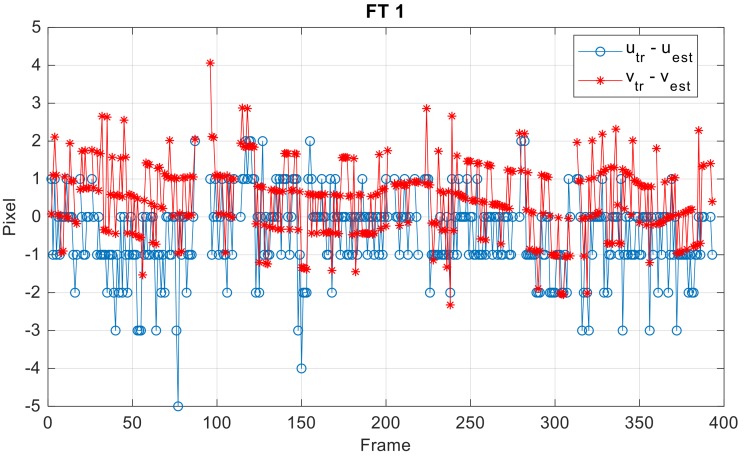
Target detection accuracy. The correlation thresholds are set to 0.86, and *τ_upd_* is set to 0.87.

With regards to the accuracy achieved by the detection and tracking architecture (with reference to the results in Table 5), the error between the true and estimated position of the target in the image plane is shown in Figure 16 for the FT 1, while a statistical analysis is presented in Table 7 for the three FTs. The detection accuracy is of the order of one pixel for all the collected data.

As discussed in Section 4.2, during the tracking process, the target position may be estimated by different techniques. Hence, it is interesting to show how successes and failures of the algorithm are distributed between Template Matching (TM) and Morphological Filtering (MF). Let us consider the FT 1 and the setting parameters used to obtain the performance shown in Table 5. The distribution of action between TM and MF is represented by the data in Table 8.

Looking at the third row, it is interesting to see that the target declaration decision is taken inside the TM search area for almost 90% of the analyzed frame. This further demonstrates the efficiency of the adopted aiding strategy based on navigation hint (as already stated at the beginning of this section). Another interesting aspect to notice is that one over four successes are ensured by the customized algorithm based on the morphological filtering operator. This aspect validates the idea of combining TM and MF to increase the robustness of detection and tracking process.

## 6. Conclusions

An original approach to detect and track a cooperative, target UAV using a camera onboard a tracker UAV is presented in this paper. The proposed algorithms exploit the concepts of template matching and morphological filtering. Furthermore, the image processing chain is augmented by navigation hints, available thanks to the cooperative nature of the formation, to predict the target position in the image plane. This strategy allows limiting the computational load, as well as the occurrence of false positives and false negatives, even when dealing with significant changes in target range, illumination condition and local background. These algorithms are also designed to be integrated within two navigation architectures developed by the authors in previous works to enable (i) high-accuracy attitude estimation for UAVs flying under nominal GNSS coverage and (ii) safe autonomous navigation for UAVs flying in GNSS-challenging environments. A flight test campaign is carried out to assess the algorithms’ performance using two customized multi-rotor UAVs. Specifically, data from three flight tests are processed to analyze achievable performance in terms of detection accuracy and success rate, as well as the sensitivity of the proposed approach deriving from the variation of its operational parameters. The target position is estimated with an accuracy of the order of one pixel, while the success rate (i.e., number of correct decisions) can be kept in the range of 85–95%. With regards to this latter aspect, different strategies to select the operational parameters properly to optimize the number of correct detections while simultaneously keeping false alarms and wrong detections close to zero are presented and motivated.

Future works will be aimed at applying the concept of cooperation considering the possibility of exploiting different image processing techniques such as deep learning-based ones, which look particularly promising for scenarios characterized by a complex non-homogenous background.

## Figures and Tables

**Table 1:** Summary of the overview on related work.

Method	Image Processing Functions		Notes
	Detection	Tracking	
[29]	Trained cascade classifier based on FAST features	N/A	A UAV-UAV distance estimation method is included
[31]	N/A	Feature matching (FAST), optical flow, local geometric filter	An additional outlier removal strategy is included (local outlier factor module)
[32]	Background motion compensation and optical flow	Kalman filtering	False alarms arising from the background motion are limited
[33]	Deep learning	N/A	Detection against a complex background is enabled
Our method	Template matching	Template matching, morphological filtering	Cooperation is exploited by aiding image processing based on the exchange of navigation data

**Table 2:** Overview of the three FTs.

FT	*N*	*N_IN_*	*N_OUT_*	*ρ* Mean (m)	*ρ* Standard Deviation (m)	Number of 360° Turns	Δ*u* Mean (pixel)	Δ*v* Mean (pixel)
**1**	398	376	22	84.2	17.1	6	38.3	24.5
**2**	361	319	42	105.9	19.5	7	29.6	25.8
**3**	381	323	58	120.2	19.5	3	33.0	21.2

**Table 3:** Statistical analysis of the error in predicted target position to carry out detection.

FT	*u_tr_*–*u_pr_* (pixel)		*v_tr_*–*v_pr_* (pixel)	
	Mean	Standard Deviation	Mean	Standard Deviation
**1**	325.2	235.0	−4.3	29.2
**2**	383.7	448.8	−16.8	67.1
**3**	247.0	300.7	−5.3	20.6

**Table 4:** Statistical analysis of the error in predicted target position to carry out tracking.

FT	*u_tr_*–*u_pr_* (pixel)		*v_tr_*–*v_pr_* (pixel)	
	Mean	Standard Deviation	Mean	Standard Deviation
**1**	1.4	28.3	−0.7	11.7
**2**	1.6	14.3	−0.1	10.5
**3**	−0.8	13.5	0.3	11.1

**Table 5:** Performance parameters for the overall detection and tracking process. The correlation thresholds are set to 0.86, and *τ_upd_* is set to 0.87.

FT	MD (%)	FA (%)	CD (%)	WD (%)	*N_upd_*	FA + WD (%)
**1**	4.27	0	95.73	0	4	0.00
**2**	10.80	1.66	87.53	0	11	1.66
**3**	23.88	0.79	75.07	0.26	20	1.05

**Table 6:** FT 3. Effect of *τ_upd_* on the performance parameters for the overall detection and tracking process. The correlation thresholds are set to 0.81.

*τ_upd_*	MD (%)	FA (%)	CD (%)	WD (%)	*N_upd_*	FA + WD (%)
0.86	15.22	2.10	82.15	0.52	35	2.62
0.87	10.24	5.77	79.53	4.46	61	10.23
0.88	14.96	2.10	82.68	0.26	65	2.36
0.89	13.91	2.36	83.20	0.52	69	2.88
0.90	12.34	2.10	85.04	0.52	82	2.62

**Table 7:** Statistical analysis of the error in estimated target position. The correlation thresholds are set to 0.86, and *τ_upd_* is set to 0.87.

FT	*u_tr_*–*u_pr_* (pixel)		*v_tr_*–*v_pr_* (pixel)	
	Mean	Standard Deviation	Mean	Standard Deviation
**1**	−0.5	1.1	0.4	1.0
**2**	−0.7	0.9	0.8	0.8
**3**	−1.3	1.6	1.0	1.5

**Table 8:** FT 1. The correlation thresholds are set to 0.86, and *τ_upd_* is set to 0.87. The distribution of action between TM inside the search area (TM IN), TM outside the search area (TM OUT) and Morphological Filtering (MF).

Distribution of action when the target is inside the FOV (376 frames)	**MD (%)**	**CD TM IN (%)**	**CD TM OUT (%)**	**CD MF (%)**	**WD TM IN (%)**	**WD TM OUT (%)**	**WD MF (%)**
	4.5	60.4	9.8	25.3	0	0	0
Distribution of action when the target is outside the FOV (22 frames)	**CD (%)**	**FA TM IN (%)**	**FA TM OUT (%)**	**FA MF (%)**			
	100	0	0	0			
Distribution of action for all the 398 frames	**TM IN (%)**	**TM OUT (%)**	**MF (%)**				
	63.2	10.3	26.5				
Distribution of action when the target is declared out (39 frames)	**MD (%)**	**CD (%)**					
	43.6	56.4					
